# Flow assignment model for quantitative analysis of diverting bulk freight from road to railway

**DOI:** 10.1371/journal.pone.0182179

**Published:** 2017-08-03

**Authors:** Chang Liu, Boliang Lin, Jiaxi Wang, Jie Xiao, Siqi Liu, Jianping Wu, Jian Li

**Affiliations:** School of Traffic and Transportation, Beijing Jiaotong University, Beijing, China; Beihang University, CHINA

## Abstract

Since railway transport possesses the advantage of high volume and low carbon emissions, diverting some freight from road to railway will help reduce the negative environmental impacts associated with transport. This paper develops a flow assignment model for quantitative analysis of diverting truck freight to railway. First, a general network which considers road transportation, railway transportation, handling and transferring is established according to all the steps in the whole transportation process. Then general functions which embody the factors which the shippers will pay attention to when choosing mode and path are formulated. The general functions contain the congestion cost on road, the capacity constraints of railways and freight stations. Based on the general network and general cost function, a user equilibrium flow assignment model is developed to simulate the flow distribution on the general network under the condition that all shippers choose transportation mode and path independently. Since the model is nonlinear and challenging, we adopt a method that uses tangent lines to constitute envelope curve to linearize it. Finally, a numerical example is presented to test the model and show the method of making quantitative analysis of bulk freight modal shift between road and railway.

## Introduction

In recent years, road transport made great contribution to our society. However, some negative effects such as air pollution, energy consumption and so on also emerged meanwhile. Hence, some administrators and scholars are considering diverting some freight flow on road to other transportation modes. Compared to road transport, railway and water transport are better choices for developing green transportation because of their low energy consumption and carbon emissions. Therefore, freight modal shift from road to rail is a potential means by which the negative effects of transport can be reduced [1].

In 2011, the European Union stated that 30% of road freight over 300 km should shift to other modes such as rail or waterborne transport by 2030, and more than 50% by 2050 [2]. At the end of 2015, the Ministry of Transport of China also recommended that railways and waterways should undertake more freight transportation [3]. However, for inland transportation, the capacity of inland waterways is usually limited. Thus, railway should take over most of the freight from road.

Generally speaking, bulk freight is more suitable for being transported by railway. Nonetheless, there is more bulk freight transported by road. For instance, China in the past several years, has transported more than 100 million tons of coal long-distance by truck from western to eastern China. Therefore, diverting bulk freight from road to parallel railway is an important strategy to reduce carbon emissions caused by the transportation industry.

To study the measures for diverting bulk freight from road to railway, the flow distribution on road and railway network under certain circumstances and the factors which will influence the distribution should first be obtained. The administrator can then design the measures based on the factors and how the factors influence the distribution. Simulating the flow distribution by means of flow assignment is an important foundation of studying the plan for adjusting the flow between road and railway. Thus, this paper studies the flow assignment problem on road and railway network from the perspective of shippers’ choosing transport mode and path. With this knowledge, a flow assignment model is constructed which considers handling cost, transfer cost, congestion cost, and capacity limitation. This model will provide theoretical support for related administrators as well as obtain the flow distribution on road and railway network under various circumstances.

## Literature review

Assigning freight flow on railway and road network involves two important problems, one is freight transport mode choice behavior and the other one is intermodal freight flow routing problem. In terms of the mode choice behavior problem, research on calculating the general costs and analysis of the related factors were made[4–6]. In addition, measures or policies for adjusting freight flow between road and railway are studied based on freight mode choice behavior [7–9]. The related achievements set great foundation for further quantitative research. Then, disaggregate models were applied to the problem. Wang et al. [10] investigated unobserved factors influencing freight mode choices between road and railway. They developed binary probit and logit models to compare the modal behavior and to verify the differences of mode choice behavior. Lian et al. [11] employed multinomial logit model and latent class model to investigate customers’ express service choice behavior, using data from a SP survey.

Besides, many researchers devoted themselves to the intermodal freight flow routing problem. He [12] proposed a directional multi-phase labeling algorithm to deal with the shortest path cost problem in container road-rail multimodal transportation. The problem considers freight collection, transfer and delivery. Wang et al. [13] formulated the multi-modal express shipments network routing problem with an arc-path model and solved it by Lingo. Yin et al. [14] analyzed the user preferences in freight transport route choice and then proposed an improved shortest path method for assigning multi-commodity transportation flows on multimodal networks. Santos et al. [15] discussed the impact of freight transport policies by means of flow assignment aiming to promote railroad intermodal transport in Europe. In recent years, low carbon transportation attracts more and more attention. Thus, the cost of carbon emissions is taken into consideration when optimizing the intermodal transportation system. Sun and Lang [16] developed a node-arc-based multi-commodity model to deal with the freight routing problem which considers schedule-based rail services, time-flexible road services and carbon dioxide emissions. Based on the carbon-reduction policy, Chen et al. [17–19] studied the optimization of mode selection for the intermodal transportation, diverting freight flow between road and railway, and integrated optimization of flow assignment in inland transportation system. Li et al. [20] presents a road truck routing problem under the carbon emission trading mechanism.

The existing achievements made great contribution to the intermodal transportation such as the methods of establishing multimodal transportation network model, the algorithm for general shortest path problem and so on. However, as far as we know, most of the existing achievements which focus on freight flow problem on road and railway network set the travel costs of all the links as constants. This method will simplify the problem, but some characteristics of the traffic flow may be omitted. In fact, to assign freight flow on the network contains road and railway, both the characteristics of road traffic flow and railway traffic flow should be taken into consideration. In terms of road traffic flow, previous study has got many achievements. Among them the BPR function is widely used to describe the relationship between the travel time and the load of the road section. It shows that the travel time on the section will increase as the load of the section increases[21]. However, the characteristic of railway freight flow is quite different. Trains are running on railway lines according to the train diagram. The travel time of a train through a railway section is set constant in advance and it will not be influenced by the load of the railway section. Besides, Shi and Li [22] proposed the concept of merging paths for the rail car path problem. Lin et al. [23, 24] pointed out that the railway freight flow assignment should obey the tree-shaped path principle and formulated this characteristic. A heuristic algorithm for the set of available paths connecting O-D pairs in a network is also proposed.

Therefore, in order to describe the freight flow assign problem on road and railway network accurately, the changeable travel time on road link, the constant travel time on railway link and related principles of railway freight flow management should be taken into consideration simultaneously because all the factors will influence the flow assignment. Thus, this paper adds these factors into the model to make the model more practical.

The remainder of this paper is organized as followed: Section 3 describes the freight flow assignment problem on road and railway network in detail and establishes a general network. Section 4 formulates the model based on the general network and analyzes the model briefly. Section 5 puts forward the method to solve the model, including preprocessing and model linearization. Lastly, a numerical example and sensitivity analysis are made in section 6 and section 7 is the conclusion.

## Problem description

There are two principles of traffic flow assignment. One is user equilibrium and the other one is system optimization. Shippers usually can choose transportation mode independently. In other words, all the shippers choose the best plan for themselves. Therefore, to simulate the flow distribution practically, the user equilibrium principle is adopted in this paper. Besides, most shippers mainly focus on the time consumption and monetary expenditure when choosing transportation plan. Thus, general cost functions which involve these two factors should be employed to represent the weights of the links or arcs in the network.

**Fig 1:**
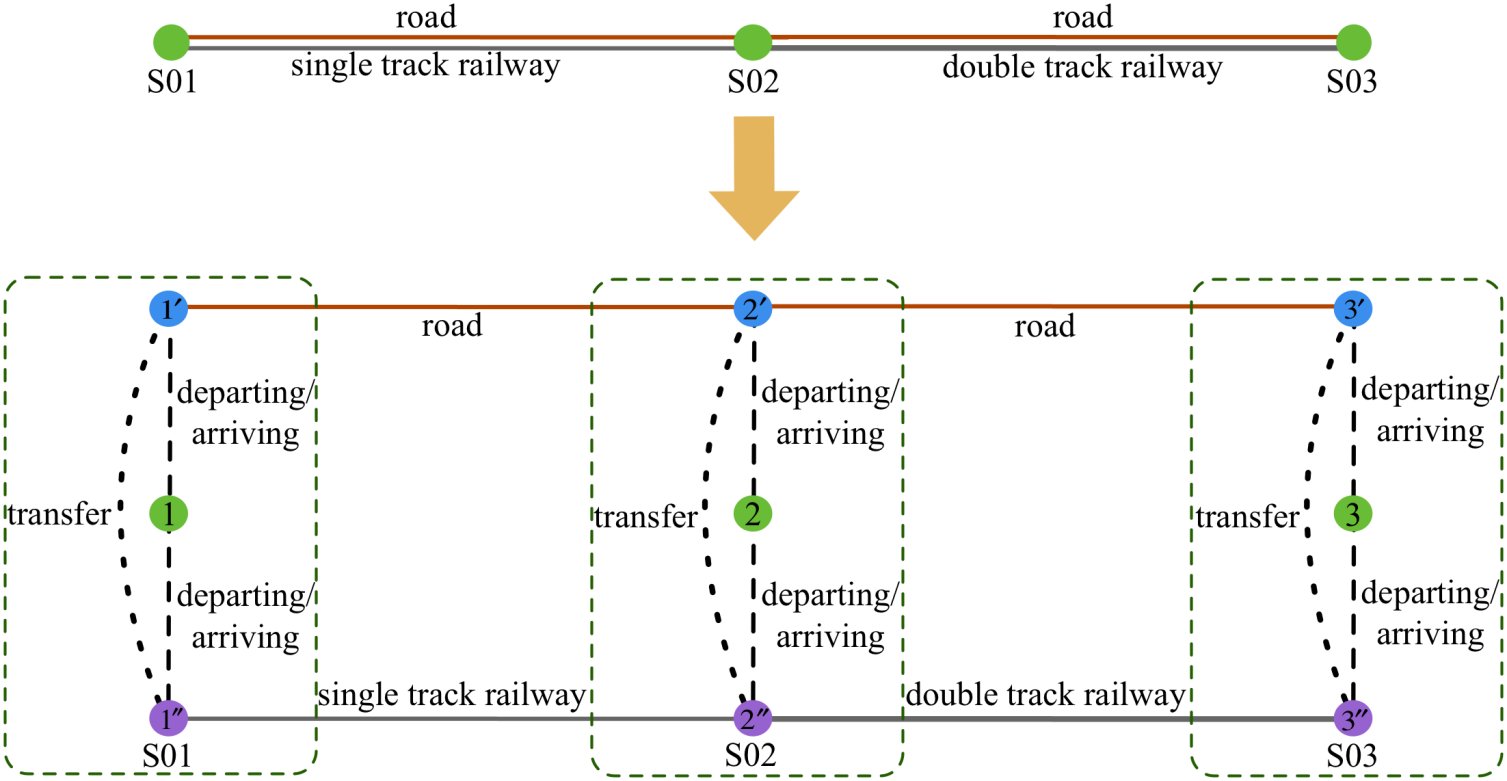
Illustration of adding virtual link.

The process of departing (including loading and waiting), arriving (including unloading and waiting) and transferring (including waiting) will also consume time and expense. Although these processes do not generate displacement, they should also be considered in the flow assignment. Thus, we add virtual links to the physical network to represent the processes. For example, in a simple network which contains three nodes, parallel roads and railways, the method of adding virtual links is shown in Fig 1.

In Fig 1, each node in the physical network is separated into three sub-nodes and the three sub-nodes are connected by virtual links which represent handling (departing and arriving) or transferring. The network that contains the virtual links is known as “general network”. In the “general network”, the green discs are the origins or destinations of the bulk freight. The blue discs are the nodes of road network and the purple discs are the nodes of railway network.

When there is a freight flow being transported by train from S01 to S03 on the physical network, the path of the freight flow on the corresponding “general network” is 1 → 1″ → 2″ → 3″ → 3. When the freight flow is transported by trunk from S01 to S02 first and then transported by train from S02 to S03, its path on the “general network” is 1 → 1′ → 2′ → 2″ → 3″ → 3. If station S02 cannot transfer freight between road and railway directly, the path will be 1 → 1′ → 2′ → 2 → 2″ → 3″ → 3. This means that the freight will first be unloaded from one mode at S02 and then loaded to the other mode.

**Fig 2:**
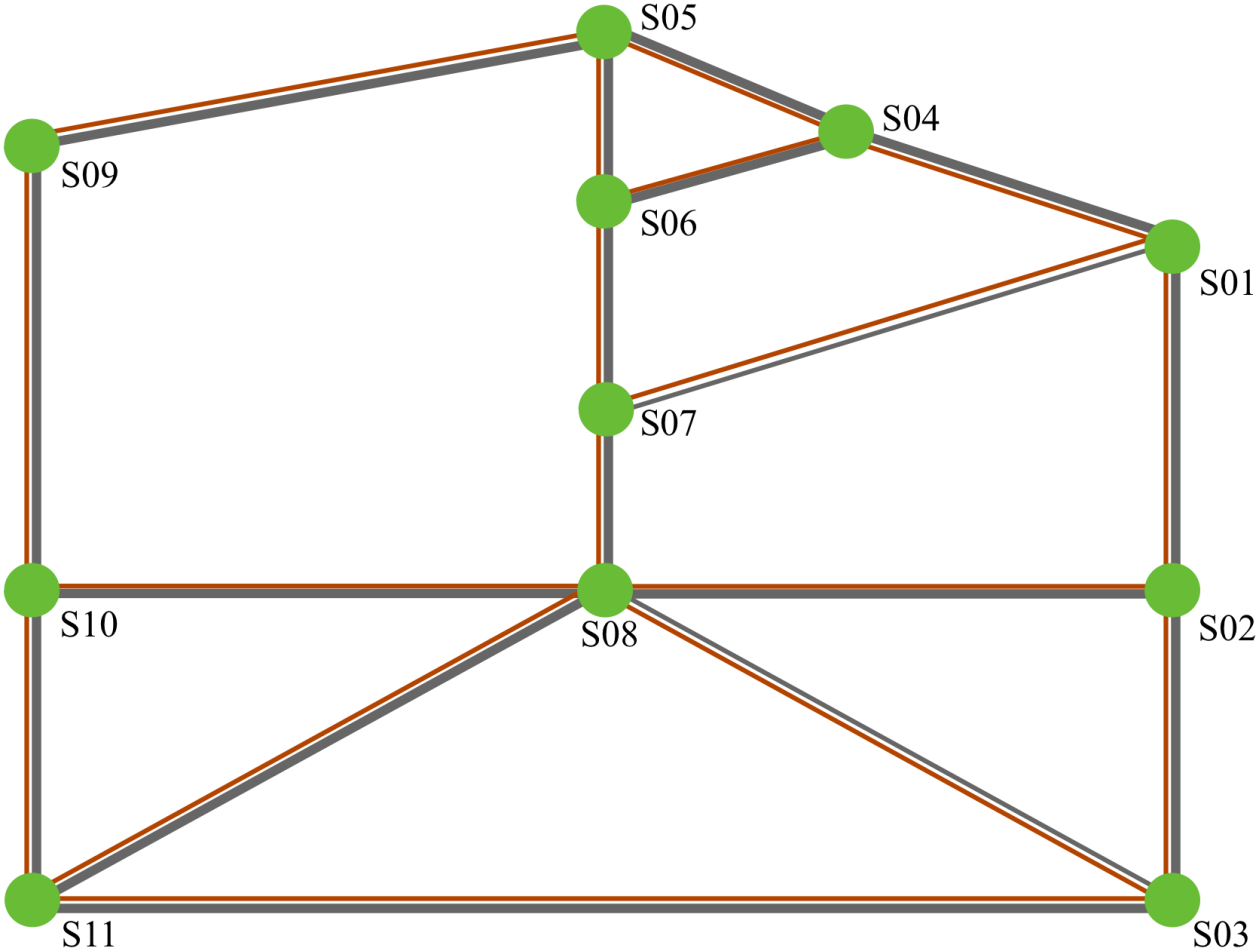
Illustration of physical network.

**Fig 3:**
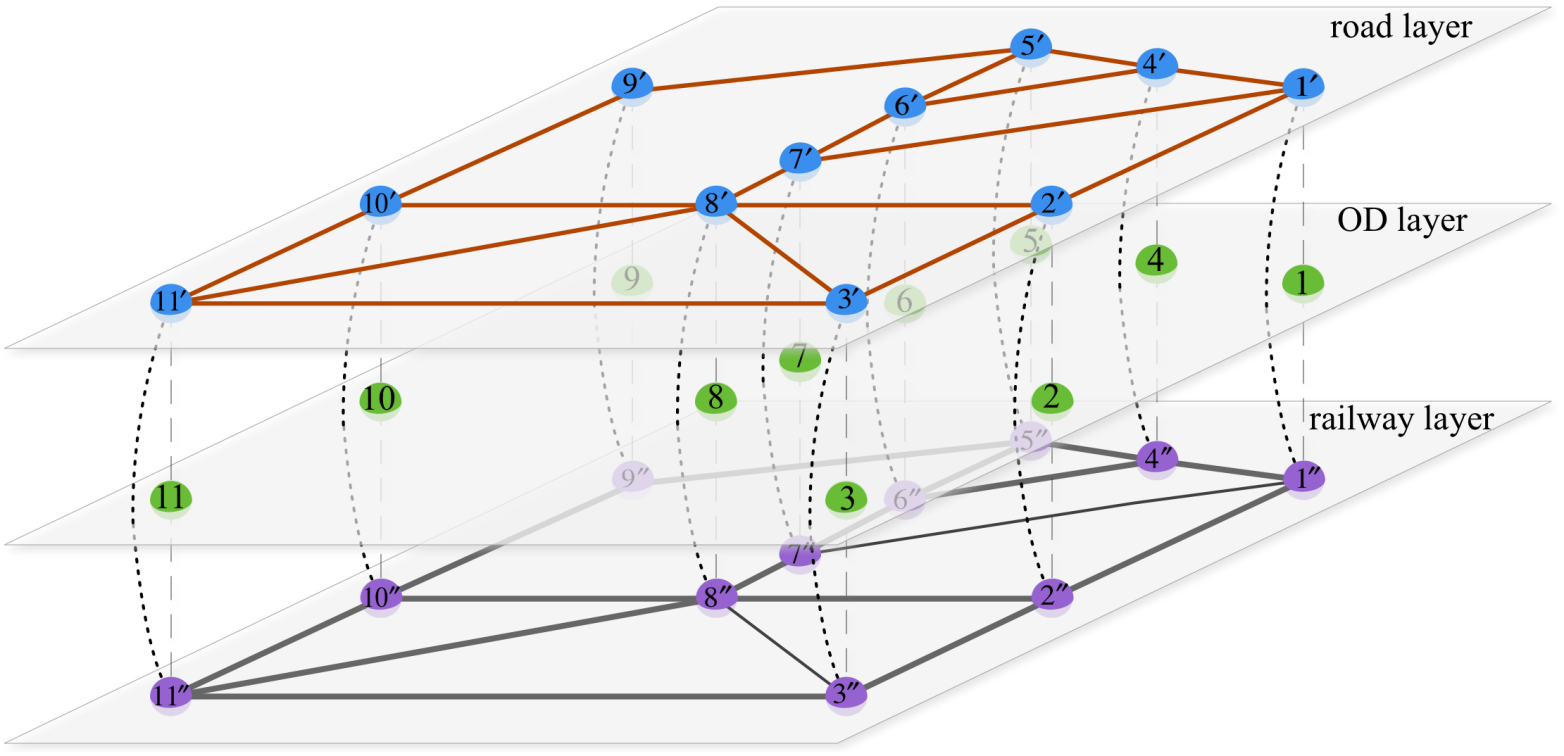
Illustration of general network.

Assigning freight flow on “general network” can take the cost of loading, unloading and transferring into consideration and make the simulated flow distribution more practical. In terms of a physical network (Fig 2), its corresponding “general network” is shown in Fig 3. Elements in Figs 2 and 3 have the same meaning as those in Fig 1.

**Fig 4:**
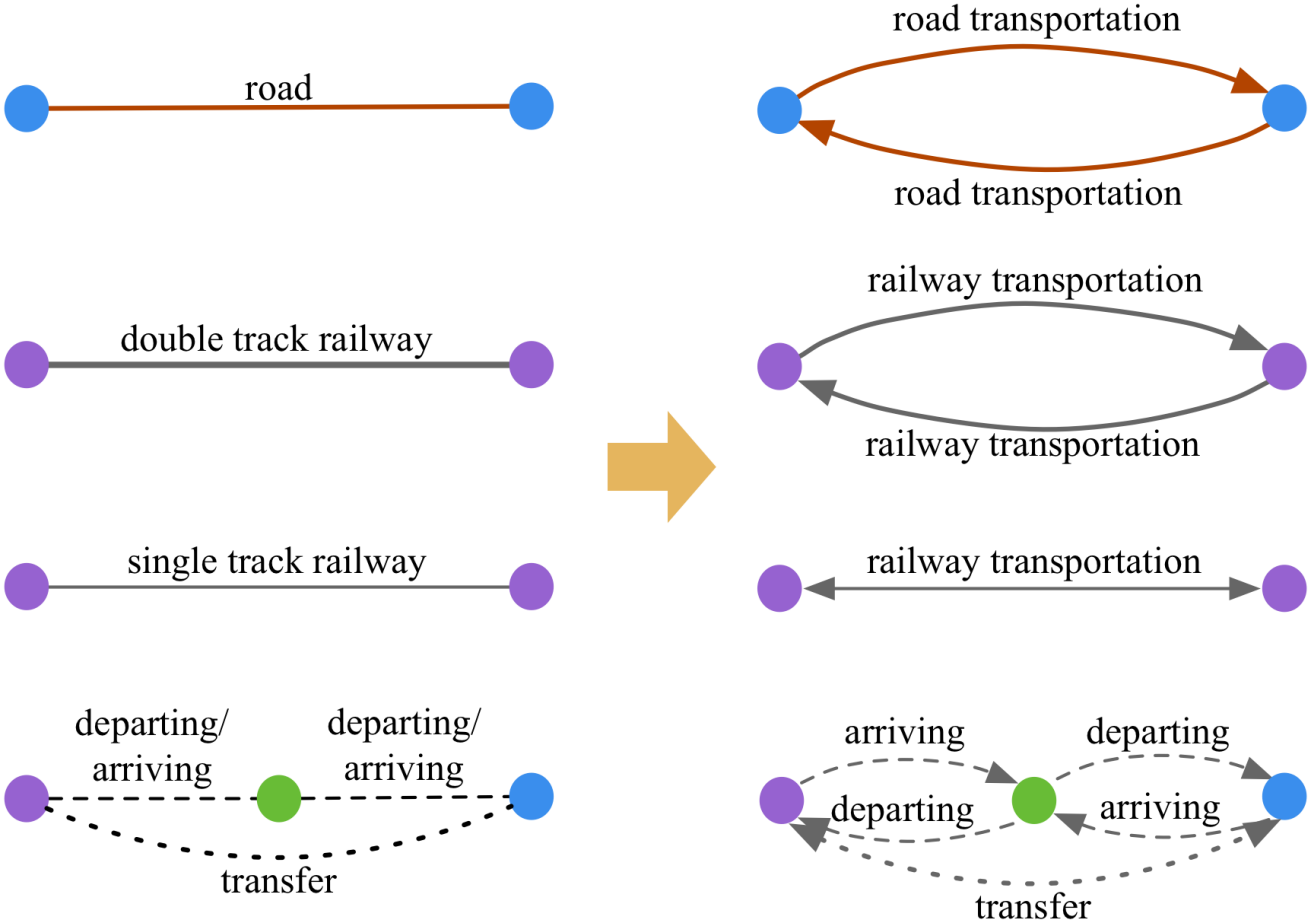
Illustration of converting links into arcs.

In Fig 3, for double track railway and road, traffic flow can run through a link in opposite directions without interfering with each other. In other words, freight flow of opposite directions can go through the same link at the same time. Therefore, this type of link is regarded as two arcs of opposite directions. However, for single track railway, only freight flow of one direction can go through a link at a certain time. Thus, if the volume of freight flow in one direction increases, the capacity of the other direction decreases. Hence, this type of link is regarded as one double-direction arc. In terms of the links represent handling (departing and arriving), the related facilities for loading and unloading are usually different and they can work independently. Thus, this type of link can be regarded as two arcs. Nevertheless, the facilities for transferring usually can transfer freight both from train to trunk and from trunk to train. Thus, the transferring link should be regarded as one arc. Before formulating the model, it is important to note that the links should be converted into arcs as Fig 4.

In Fig 4, the weights of the arcs are calculated by the general cost functions which consider time and monetary expenditure. The general cost function will be introduced in the next section. In the subsequent model formulation, all the relative parameters will be set based on arcs.

## Model formulation

### Notations

The sets and parameters in the model are listed as follows.

### Sets

*V*: The set of nodes on OD layer.
*E*: The set of all the arcs. *E* = *E*^road^ ∪ *E*^rail^∪ *E*^handling^ ∪ *E*^transfer^, *E*^road^, *E*^rail^, *E*^handling^ and *E*^transfer^ represent, respectively, the set of road transportation arcs, railway transportation arcs, handling arcs and transferring arcs. All the arcs are indexed by *m*.
*P*(*i*, *j*): The set of available paths from *i* to *j*. The paths in the set are indexed by *p*.

### Parameters

*q*_*ij*_: The bulk freight transportation demand from *i* to *j*.
$\delta_{ij}^{pm}$
The path-arc correlation parameter. If arc *m* is on path *p* from *i* to *j*, $\delta_{ij}^{pm}=1$. Otherwise, $\delta_{ij}^{pm}=0$.
*F*_*m*_: The total volume of bulk freight that go through arc *m*, measured in million ton/year.
*e*^road^: The expenditure of transporting 1 ton of bulk freight for 1 kilometer on road. Note that the expenditure mentioned in this paper means all the monetary expense including tax and so on.
*e*^rail^: The expenditure of transporting 1 ton of bulk freight for 1 kilometer on railway.
*e*_*m*_: The expenditure of loading, unloading or transferring 1 ton of bulk freight on arc *m* (*m* ∈ *E*^handling^ ∪ *E*^transfer^).
*θ*: The time value parameter of bulk freight.
*μ*: The coefficient for transforming the volume of bulk freight measured by ton into that measured by Passenger Car Unit (PCU).
*l*_*m*_: The physical length of arc *m* (*m* ∈ *E*^road^ ∪ *E*^rail^). In terms of ∀*m* ∈ *E*^handling^ ∪ *E*^transfer^, *l*_*m*_ = 0.
*v*_*m*_: The travel speed on arc *m*. For ∀*m* ∈ *E*^road^, it represents the travel speed of trunk on arc *m* without any interference. For ∀*m* ∈ *E*^rail^, it represents the regulation average travel speed of freight train on arc *m*.
*t*_*m*_: The shortest time consumption of arc *m*. For ∀*m* ∈ *E*^road^ ∪ *E*^rail^, $t_{m}=\frac{l_{m}}{v_{m}}$. For ∀*m* ∈ *E*^handling^ ∪ *E*^transfer^, it represents the time consumption of corresponding process.
*c*_*m*_(*F*_*m*_): The general cost function of arc *m*.
*c*_*m*_(*F*_*m*_): The primitive function of *c*_*m*_(*F*_*m*_).
*b*_*m*_: The capacity of arc *m*.
*M*: A sufficient large number.
*O*_*m*_: The existing load of arc *m*. For ∀*m* ∈ *E*^road^, it represents the quantity of other cars on arc *m* which is measured in million PCU/year. For ∀*m* ∈ *E*^handling^ ∪ *E*^transfer^, it represents the load of other freight on arc *m* which is measured in million ton/year. For ∀*m* ∈ *E*^rail^, it represents the equivalent freight volume of other trains which is measured in million ton/year. The equivalent freight volume is converted according to the capacity occupied by the train.
*α*, *β*: Parameters of BPR function.

### Decision variable

$f_{ij}^{p}$
The volume of freight flow which is from *i* to *j* and go through path *p*.

### Formulation of general cost function

#### General cost function of road transportation

In terms of road transportation, the time consumption caused by congestion is an important factor which influences shippers’ choice of path. Thus, the relationship between travel time and the load of the road transportation arc is a necessary part of the general cost function. To formulate this function, many scholars are devoted to related research. Among the achievements, the function developed by Bureau of Public Road in the U.S. is a famous one. It is formulated as follows.

$$t_{m}\left(F_{m}\right)=t_{m}\left[1+\alpha {\left(\frac{F_{m}}{b_{m}}\right)}^{\beta }\right]$$ (1)

Formula (1) means that the travel time on link *m* increases as the load of the link growing. When the load exceeds the capacity, the travel time will increase sharply. This can reflect the time consumption caused by congestion.

Since the monetary expenditure should also be involved in the general cost function, the corresponding item should be added to the function. Also, the time value parameter should be employed to transfer the time consumption into monetary expenditure. The general cost function for road transportation is shown as follows.

$$c_{m}\left(F_{m}\right)=e^{\text{road}}l_{m}+\theta t_{m}\left[1+\alpha {\left(\frac{O_{m}+\mu F_{m}}{b_{m}}\right)}^{\beta }\right]\;\forall m\in E^{\text{road}}$$ (2)

We should note that *F*_*m*_ is measured in tons. However, the load of road is always measured in PCU, thus parameter *μ* is used for unifying the unit of measurement.

#### General cost function of handling and transferring

The quantity of workers and handling facilities is constant and the freight will not interact when being handled. Consequently, the handling efficiency of a station is invariable within its capacity. The general cost function is shown as follows

$$c_{m}\left(F_{m}\right)=e_{m}+\theta t_{m}\;\forall m\in E^{\text{handling}}\cup E^{\text{transfer}}$$ (3)

with the following constraint.

$$F_{m}+O_{m}\leq b_{m}\;\forall m\in E^{\text{handling}}\cup E^{\text{transfer}}$$ (4)

However, there should not be a capacity constraint in the user equilibrium model because it may influence the optimal solution. Therefore, the constraint should be integrated into the function. Here we adopt penalty item instead of adding constraints.

$$c_{m}\left(F_{m}\right)=e_{m}+\theta \left[t_{m}+{\left(\frac{F_{m}+O_{m}}{b_{m}}\right)}^{M}\left(F_{m}+O_{m}-b_{m}\right)\right]\;\forall m\in E^{\text{handling}}\cup E^{\text{transfer}}$$ (5)

In the above formula, when *F*_*m*_ + *O*_*m*_ ≤ *b*_*m*_ namely the load on the link is within the capacity of the arc, *c*_*m*_(*F*_*m*_ ≈ *e*_*m*_ + *θt*_*m*_); when *F*_*m*_ + *O*_*m*_ > *b*_*m*_ namely the load on the link beyond the capacity of the arc, *c*_*m*_(*F*_*m*_)→ ∞. Under the condition that the model is to minimize the objective function, theoretically, the model will make *F*_*m*_ + *O*_*m*_ − *b*_*m*_ → 0 in the solving progress when *F*_*m*_ + *O*_*m*_ > *b*_*m*_. This can equivalently meet the capacity constraint.

#### General cost function of rail transportation

The railway transportation is organized according to the train diagram. In the train diagram, the travel time in each railway section is set as constant. Thus, in the general network, the time consumption of each railway transportation arc is a constant which depends on the train diagram and has no relationship with the load of the arc. In addition, the capacity of each railway section is limited. When the capacity of a certain section is fully used, no more trains will be arranged in this section. Therefore, there should also be capacity constraint in the formulation. Referring to the method mentioned above, the general cost function of rail transportation arc is shown as follows.

$$c_{m}\left(F_{m}\right)=e^{\text{rail}}l_{m}+\theta \left[t_{m}+{\left(\frac{F_{m}+O_{m}}{b_{m}}\right)}^{M}\left(F_{m}+O_{m}-b_{m}\right)\right]\;\forall m\in E^{\text{rail}}$$ (6)

### Model formulation and analysis

According to the analysis of general network and the essential elements of user equilibrium model, the general network based bulk freight flow assignment model is formulated as follows.

$$\text{M}1\;\text{min}Z\left(F\right)=\sum_{m\in E}\int_{0}^{F_{m}}c_{m}\left(\xi \right)d\xi$$ (7)

$$\text{s}.\text{t}.\;\sum_{p\in P\left(i,j\right)}f_{ij}^{p}=q_{ij}\;\forall i\in V,\forall j\in V$$ (8)

$$f_{ij}^{p}\geq 0\;\forall i\in V,\forall j\in V,\forall p\in P\left(i,j\right)$$ (9)

In M1, $F_{m}=\sum_{i\in V}\sum_{j\in V}\sum_{p\in P\left(i,j\right)}f_{ij}^{p}\delta_{ij}^{pm}$. *ξ* is the independent variable. The objective function is the sum of the results of integrating the general cost functions. The model contains two constraints. The first one means that, for each OD pair, the sum of the loads on all the paths should be equal to the volume of the OD demand. The second one means that the decision variables should be non-negative. The objective function has no practical meaning. However, it can be demonstrated that the optimal solution of M1 is equivalent with user equilibrium [21].

To analyze the character of M1, we transform the objective function into the detailed form.

$$\int_{0}^{F_{m}}c_{m}\left(\xi \right)d\xi ={C_{m}\left(\xi \right)|}_{0}^{F_{m}}$$ (10)

Thus, for ∀*m* ∈ *E*^road^ the corresponding item can be transformed as follows.

$$\int_{0}^{F_{m}}c_{m}\left(\xi \right)d\xi =e^{\text{road}}l_{m}F_{m}+\theta t_{m}F_{m}+\frac{\theta t_{m}\alpha b_{m}}{\mu \left(\beta +1\right)}{\left(\frac{O_{m}+\mu F_{m}}{b_{m}}\right)}^{\beta +1}-\frac{\theta t_{m}\alpha b_{m}}{\mu \left(\beta +1\right)}{\left(\frac{O_{m}}{b_{m}}\right)}^{\beta +1}$$ (11)

In Formula (11), $-\frac{\theta t_{m}\alpha b_{m}}{\mu \left(\beta +1\right)}{\left(\frac{O_{m}}{b_{m}}\right)}^{\beta +1}$ is a constant item which has no influence on the optimizing process, so it can be omitted. Thus, the item can finally be transformed as Formula (12).

$$e^{\text{road}}l_{m}F_{m}+\theta t_{m}F_{m}+\frac{\theta t_{m}\alpha b_{m}}{\mu \left(\beta +1\right)}{\left(\frac{O_{m}+\mu F_{m}}{b_{m}}\right)}^{\beta +1}$$ (12)

$$\forall m\in E^{\text{handling}}\cup E^{\text{transfer}}$$

$$\int_{0}^{F_{m}}c_{m}\left(\xi \right)d\xi =e_{m}F_{m}+\theta t_{m}F_{m}+\left(F_{m}+O_{m}-b_{m}-\frac{b_{m}}{M+1}\right)\cdot \frac{{\left(F_{m}+O_{m}\right)}^{M+1}}{\left(M+2\right)b_{m}^{M}}$$ (13)

$$\forall m\in E^{\text{rail}}$$

$$\int_{0}^{F_{m}}c_{m}\left(\xi \right)d\xi =e^{\text{rail}}l_{m}F_{m}+\theta t_{m}F_{m}+\left(F_{m}+O_{m}-b_{m}-\frac{b_{m}}{M+1}\right)\cdot \frac{{\left(F_{m}+O_{m}\right)}^{M+1}}{\left(M+2\right)b_{m}^{M}}$$ (14)

Per the above analysis, the objective function can be divided into three parts. All the parts are non-linear items. Therefore, M1 is a non-linear model.

## Solution approach

### Preprocessing

M1 is an arc-path model. The most important preprocess of arc-path model is to generate the sets of available paths between the OD pairs (the sets are denoted as *P*(*i*, *j*)) and obtain the values of path-arc correlation parameters (the parameters are denoted as $\delta_{ij}^{pm}$). In terms of a single modal network, the available paths can be generated by using *k*-shortest algorithm. However, for a multimodal network which contains railway and road, some constraints should be considered when searching for the paths.

Since the truck drivers can choose their path independently, all the paths on road network which connecting the specified nodes can be chosen as available path. However, in a rail system, train drivers cannot choose the path freely. All the freight trains must go along specified paths. In addition, because of the special character of railway operation, the railway freight flow paths should obey the following two important rules: 1) For each OD pair, only one path can be chose; and 2) if two or more shipments to the same destination converge at a railway yard, they will be merged and considered as one flow and run on the same path during the remainder of the trip even if they may come from different origins [24]. Therefore, if a freight flow is transported on road, there are multiple available paths that can be chosen. If the freight flow is transported on railway, only one path can be chosen. If the freight flow is transported by road and railway successively, it can choose multiple paths when it is on road but only one specified path when it is on railway. This will make it very difficult to generate the set of available paths. Therefore, we designed a special method to search the available path in the “general network.” Here we assume that the bulk freight can be transferred no more than one time during its journey. The processes are shown as follows:

Step 1 Generate the railway car flow paths between any two nodes on the railway layer according to the regulations of railway car flow path issued by the railway company. The set of all the paths is denoted as *P*^rail^. The available path between *i*″ and *j*″ is denoted as *P*^rail^(*i*″, *j*″).
Step 2 Generate the available paths between any two nodes on the road layer based on the *k*-shortest path method [23]. The set of all the paths is denoted as *P*^road^. The set of available paths between *i*′ and *j*′ is denoted as *P*^road^(*i*′, *j*′).
Step 3 Select an OD pair (*i*, *j*) on the OD layer randomly. The corresponding set of available paths on the road layer is *P*^road^(*i*′, *j*′).
Step 4 Select a path *p*_*k*_ from *P*^road^(*i*′, *j*′) randomly and denote the set of in-between nodes (not including *i*′ and *j*′) on *p*_*k*_ as $S_{p_{k}}^{\prime }\left(i^{\prime },j^{\prime }\right)$. The set of corresponding nodes on railway layer is denoted as $S_{p_{k}}^{\prime \prime }\left(i^{\prime \prime },j^{\prime \prime }\right)$.
Step 5 Select a node $s_{r}^{\prime }$ as the transfer node from $S_{p_{k}}^{\prime }\left(i^{\prime },j^{\prime }\right)$ randomly and intercept the section $i^{\prime }\to s_{r}^{\prime }$ out of *p*_*k*_. The node in $S_{p_{k}}^{\prime \prime }\left(i^{\prime \prime },j^{\prime \prime }\right)$ corresponding to $s_{r}^{\prime }$ is labeled as $s_{r}^{\prime \prime }$.
Step 6 Connect $s_{r}^{\prime }\to s_{r}^{\prime \prime }$, *i*→*i*′, *j*″ → *j*, respectively.
Step 7 Call the railway transportation path of $s_{r}^{\prime \prime }\to j^{\prime \prime }$ from *P*^rail^.
Step 8 Connect the five sections described in step 5–7 and form the road-railway intermodal transportation path.
Step 9 Call the railway transportation path of $i^{\prime \prime }\to s_{r}^{\prime \prime }$ from *P*^rail^.
Step 10 Connect $s_{r}^{\prime \prime }\to s_{r}^{\prime }$, *i*″ → *i*′, *j*′ → *j*, respectively.
Step 11 Intercept the section $s_{r}^{\prime }\to j^{\prime }$ out of *p*_*k*_.
Step 12 Connect the five sections described in step 9–11 and form the railway-road intermodal transportation path.
Step 13 Select another node in $S_{p_{k}}^{\prime }\left(i^{\prime },j^{\prime }\right)$ and repeat Step 5–12.
Step 14 Repeat Step 5–13 until all the nodes in $S_{p_{k}}^{\prime }\left(i^{\prime },j^{\prime }\right)$ are traversed.
Step 15 Select another path in *P*^road^(*i*′, *j*′) and repeat Step 4–14.
Step 16 Repeat Step 4–15 until all the paths in *P*^road^(*i*′, *j*′) are traversed.
Step 17 Select another OD pair from the OD layer and repeat Step 3–16.
Step 18 Repeat Step 3–17 until all the OD pairs in the OD layer are traversed.
Step 19 Set the value of $\delta_{ij}^{pm}$ according to all the paths.

Up to now, we have obtained the available paths and the path-arc correlation parameters and became ready for solving the model.

### Linearization

M1 is a non-linear model which is very difficult to be solved by traditional method in acceptable time. Therefore, linearizing the model and then solving it by linear programming solver is a feasible choice. R. Borndörfer et al. [25] put forward a secant line based method to linearize non-linear functions. The method is very helpful to this type of problem. In our model, tangent lines are easier to obtain than secant lines. Here we introduce a tangent line based linearization method.

**Fig 5:**
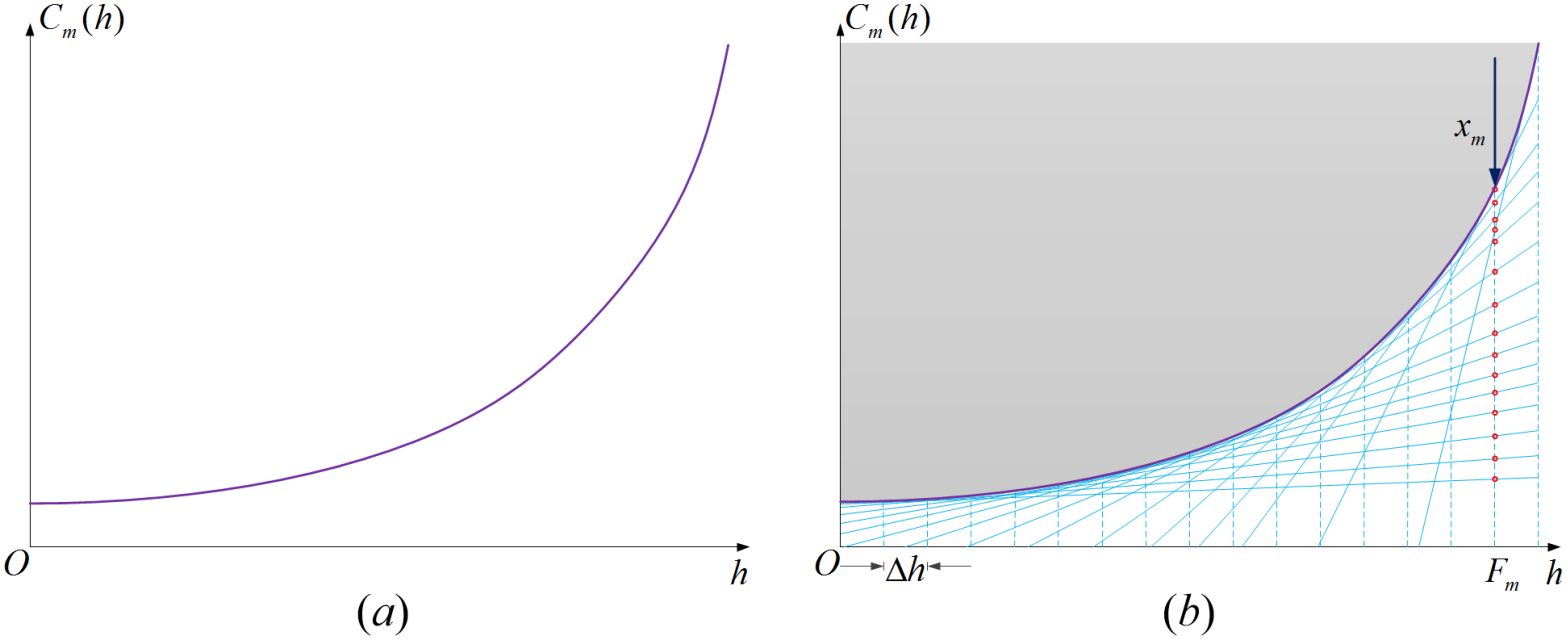
Illustration of linearizing the road transportation cost function.

For ∀*m* ∈ *E*^road^, the corresponding item in objective function is a smooth curve like that shown in Fig 5(*a*). In terms of this kind of function, we can insert *N* nodes on the curve with equal space between each other and add tangent line on each node. Then the original curve can be approximately replaced by the envelope curve formed by the tangent lines. The larger *N* is, the smaller the error between the original curve and the envelope curve will be. The illustration is shown in Fig 5(*b*).

Based on this, we introduce assistant decision variable *x*_*m*_ and formulate *x*_*m*_ and *F*_*m*_ as follows:

$$\omega_{1}^{m}\left(h\right)F_{m}+\omega_{2}^{m}\left(h\right)\leq x_{m}\;\forall m\in E^{\text{road}},\forall h=n\Delta h,n=0,1,2,\ldots ,N$$ (15)

In Formula (15), $\omega_{1}^{m}\left(h\right)=C_{m}^{\prime }\left(h\right)=c_{m}\left(h\right)$ (Here $C_{m}^{\prime }\left(h\right)$ is the derivative of *C*_*m*_(*h*)), $\omega_{2}^{m}\left(h\right)=C_{m}\left(h\right)-\omega_{1}^{m}\left(h\right)h$, Δ*h* is the space between the adjacent inserted nodes. The geometrical meaning of this constraint can be explained as follows. For any *F*_*m*_ in Fig 5(*b*), the value of *x*_*m*_ is larger than all the ordinate values of the red nodes. In other words, for any *F*_*m*_, *x*_*m*_ will always be restricted in the shadow area in Fig 5(*b*). Since the model aims at minimizing the objective function, when replacing $\sum_{m\in E^{\text{road}}}\int_{0}^{F_{m}}c_{m}\left(\xi \right)d\xi$ in the objective function with $\sum_{m\in E^{\text{road}}}x_{m}$, *x*_*m*_ will minimize its value in the solving process. In other words, the values of *x*_*m*_ will be on the lower bound of the shadow area namely on the envelope curve. Therefore, for any *F*_*m*_ (*m* ∈ *E*^road^), $\int_{0}^{F_{m}}c_{m}\left(\xi \right)d\xi$ can be replaced by *x*_*m*_. Thus, the item is linearized.

**Fig 6:**
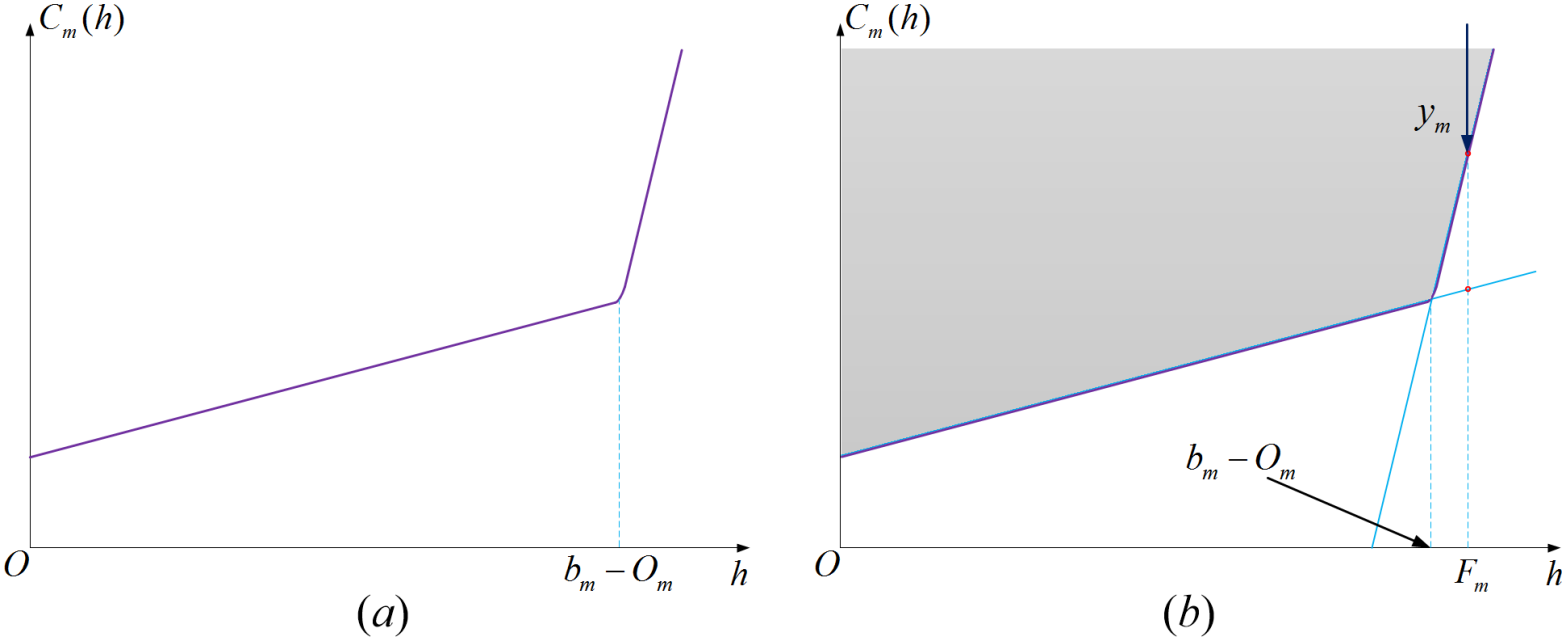
Illustration of linearizing the handling and transferring cost function.

For ∀*m* ∈ *E*^handling^ ∪ *E*^transfer^, the corresponding item in objective function is also a smooth curve which is shown in Fig 6(*a*). However, because of the effect caused by the penalty item, the curve can be regard as a broken line formed by two straight lines. The abscissa value of the inflection point is *b*_*m*_ − *O*_*m*_. Thus, the curve can be approximately replaced by the envelope curve formed by two straight lines. The illustration is shown in Fig 6(*b*). In Formula (13), when *F*_*m*_ + *O*_*m*_ ≤ *b*_*m*_,$\left(F_{m}+O_{m}-b_{m}-\frac{b_{m}}{M+1}\right)\cdot \frac{{\left(F_{m}+O_{m}\right)}^{M+1}}{\left(M+2\right)b_{m}^{M}}\to 0$, and when *F*_*m*_ + *O*_*m*_ > *b*_*m*_,$\left(F_{m}+O_{m}-b_{m}-\frac{b_{m}}{M+1}\right)\cdot \frac{{\left(F_{m}+O_{m}\right)}^{M+1}}{\left(M+2\right)b_{m}^{M}}\to \infty$. Thus, the analytic expression of the two straight lines can be formulated as:

$$C_{m}\left(h\right)=e_{m}h+\theta t_{m}h\;h\leq b_{m}-O_{m}$$ (16)

$$C_{m}\left(h\right)=e_{m}h+\theta t_{m}h+M\left(h+O_{m}-b_{m}\right)\;h>b_{m}-O_{m}$$ (17)

Therefore, by adopting the method similar with that for dealing with ∀*m* ∈ *E*^road^, we introduce assistant decision variable *y*_*m*_, construct linearized constraints and replace the corresponding items in the objective function. The geometrical meaning is shown in Fig 6(*b*).

$$e_{m}F_{m}+\theta t_{m}F_{m}\leq y_{m}\;\forall m\in E^{\text{handling}}\cup E^{\text{transfer}}$$ (18)

$$e_{m}F_{m}+\theta t_{m}F_{m}+M\left(F_{m}+O_{m}-b_{m}\right)\leq y_{m}\;\forall m\in E^{\text{handling}}\cup E^{\text{transfer}}$$ (19)

For ∀*m* ∈ *E*^rail^, the method which is similar to that for ∀*m* ∈ *E*^handling^ ∪ *E*^transfer^ can be adopted. We introduce assistant decision variable *z*_*m*_ and construct linearized constraints as follows.

$$e^{\text{rail}}l_{m}F_{m}+\theta t_{m}F_{m}\leq z_{m}\;\forall m\in E^{\text{rail}}$$ (20)

$$e^{\text{rail}}l_{m}F_{m}+\theta t_{m}F_{m}+M\left(F_{m}+O_{m}-b_{m}\right)\leq z_{m}\;\forall m\in E^{\text{rail}}$$ (21)

In conclusion, M1 can be transformed into M2 which is shown as follows.

$$\text{M}2\;\text{min}Z=\sum_{m\in E^{\text{road}}}x_{m}+\sum_{m\in E^{\text{handling}}\cup E^{\text{transfer}}}y_{m}+\sum_{m\in E^{\text{rail}}}z_{m}$$ (22)

$$\text{s}.\text{t}.\;\omega_{1}^{m}\left(h\right)F_{m}+\omega_{2}^{m}\left(h\right)\leq x_{m}\;\forall m\in E^{\text{road}},\forall h=n\Delta h,n=0,1,2,\ldots ,N$$ (23)

$$e_{m}F_{m}+\theta t_{m}F_{m}\leq y_{m}\;\forall m\in E^{\text{handling}}\cup E^{\text{transfer}}$$ (24)

$$e_{m}F_{m}+\theta t_{m}F_{m}+M\left(F_{m}+O_{m}-b_{m}\right)\leq y_{m}\;\forall m\in E^{\text{handling}}\cup E^{\text{transfer}}$$ (25)

$$e^{\text{rail}}l_{m}F_{m}+\theta t_{m}F_{m}\leq z_{m}\;\forall m\in E^{\text{rail}}$$ (26)

$$e^{\text{rail}}l_{m}F_{m}+\theta t_{m}F_{m}+M\left(F_{m}+O_{m}-b_{m}\right)\leq z_{m}\;\forall m\in E^{\text{rail}}$$ (27)

$$\sum_{p\in P\left(i,j\right)}f_{ij}^{p}=q_{ij}\;\forall i\in V,\forall j\in V$$ (28)

$$f_{ij}^{p}\geq 0\;\forall i\in V,\forall j\in V,\forall p\in P\left(i,j\right)$$ (29)

M2 is a linear programming model which can be solved by commercial software such as Gurobi, Cplex or Lingo.

## Numerical example

### Input data

This paper takes the network in Fig 2 as example to test the model. The lengths of the links in the network are shown in Table 1.

**Table 1:** The lengths of road and railway links (km).

No.	Node 1	Node 2	Length of road link	Length of railway link
**1**	S01	S02	295	281
**2**	S01	S04	193	190
**3**	S01	S07	442	444
**4**	S02	S03	424	408
**5**	S02	S08	222	225
**6**	S03	S08	418	577
**7**	S03	S11	475	511
**8**	S04	S05	174	178
**9**	S04	S06	209	178
**10**	S05	S06	121	127
**11**	S05	S09	289	323
**12**	S06	S07	174	231
**13**	S07	S08	134	124
**14**	S08	S10	281	270
**15**	S08	S11	597	651
**16**	S09	S10	408	406
**17**	S10	S11	472	521

We assume that S01, S02 and S03 are the demanders of the bulk freight. S05, S06, S07, S08, S09, S10 and S11 are the suppliers of the bulk freight. The OD matrix is shown in Table 2.

**Table 2:** OD matrix (million ton/year).

	S01	S02	S03
**S05**	5.10	8.23	3.45
**S06**	15.67	12.56	13.37
**S07**	1.02	5.43	3.21
**S08**	6.51	4.38	2.56
**S09**	6.21	11.45	15.77
**S10**	2.38	1.52	6.78
**S11**	0.00	4.21	0.00

After converting the links in Fig 3 into arcs, the information of arcs are shown in Tables 3–6 respectively.

**Table 3:** The information of road transportation arcs (million PCU/year).

*m*	endpoints	*b*_*m*_	*O*_*m*_	*m*	endpoints	*b*_*m*_	*O*_*m*_	*m*	endpoints	*b*_*m*_	*m*
**1**	1′ → 2′	28	24.56	**13**	3′ → 11′	28	24.12	**25**	5′ → 9′	28	25.77
**2**	2′ → 1′	28	25.98	**14**	11′ → 3′	28	22.54	**26**	9′ → 5′	28	21.55
**3**	2′ → 3′	28	24.75	**15**	4′ → 5′	28	25.42	**27**	8′ → 10′	28	25.43
**4**	3′ → 2′	28	22.22	**16**	5′ → 4′	28	22.45	**28**	10′ → 8′	28	22.99
**5**	1′ → 4′	28	25.65	**17**	4′ → 6′	28	25.34	**29**	8′ → 11′	28	24.04
**6**	4′ → 1′	28	22.11	**18**	6′ → 4′	28	22.44	**30**	11′ → 8′	28	25.79
**7**	1′ → 7′	28	25.54	**19**	6′ → 5′	28	24.33	**31**	9′ → 10′	28	22.31
**8**	7′ → 1′	28	22.23	**20**	5′ → 6′	28	22.22	**32**	10′ → 9′	28	24.43
**9**	2′ → 8′	28	24.34	**21**	6′ → 7′	28	22.12	**33**	10′ → 11′	28	21.88
**10**	8′ → 2′	28	22.55	**22**	7′ → 6′	28	22.44	**34**	11′ → 10′	28	25.49
**11**	8′ → 3′	28	24.76	**23**	7′ → 8′	28	21.23				
**12**	3′ → 8′	28	22.89	**24**	8′ → 7′	28	24.77				

**Table 4:** The information of handling arcs (million ton/year).

*m*	endpoints	*b*_*m*_	*O*_*m*_	*m*	endpoints	*b*_*m*_	*O*_*m*_	*m*	endpoints	*b*_*m*_	*O*_*m*_
**35**	1 → 1′	80	39.82	**50**	4″ → 4	80	39.77	**65**	8 → 8″	80	41.03
**36**	1′ → 1	80	39.57	**51**	5 → 5′	80	37.96	**66**	8″ → 8	80	59.73
**37**	1 → 1″	80	56.92	**52**	5′ → 5	80	39.77	**67**	9 → 9′	80	38.16
**38**	1″ → 1	80	55.02	**53**	5 → 5″	80	38.46	**68**	9′ → 9	80	44.52
**39**	2 → 2′	90	43.67	**54**	5″ → 5	80	37.85	**69**	9 → 9″	80	35.16
**40**	2′ → 2	90	49.18	**55**	6 → 6′	90	35.28	**70**	9″ → 9	80	39.37
**41**	2 → 2″	90	47.26	**56**	6′ → 6	90	47.96	**71**	10 → 10′	80	35.03
**42**	2″ → 2	90	55.08	**57**	6 → 6″	80	53.45	**72**	10′ → 10	80	41.92
**43**	3 → 3′	80	52.28	**58**	6″ → 6	80	39.57	**73**	10 → 10″	80	45.57
**44**	3′ → 3	80	41.93	**59**	7 → 7′	80	36.93	**74**	10″ → 10	80	51.37
**45**	3 → 3″	80	56.47	**60**	7′ → 7	80	54.37	**75**	11 → 11′	80	50.83
**46**	3″ → 3	80	42.84	**61**	7 → 7″	80	41.57	**76**	11′ → 11	80	53.94
**47**	4 → 4′	80	46.67	**62**	7″ → 7	80	37.75	**77**	11 → 11″	80	47.93
**48**	4′ → 4	80	53.37	**63**	8 → 8′	80	44.35	**78**	11″ → 11	80	44.73
**49**	4 → 4″	80	48.96	**64**	8′ → 8	80	59.24				

**Table 5:** The information of transfer links (million ton/year).

*m*	endpoints	*b*_*m*_	*O*_*m*_	*m*	endpoints	*b*_*m*_	*O*_*m*_	*m*	endpoints	*b*_*m*_	*O*_*m*_
**79**	1′—1″	60	19.99	**83**	5′—5″	60	16.78	**87**	9′—9″	60	20.78
**80**	2′—2″	60	20.21	**84**	6′—6″	60	23.33	**88**	10′—10″	60	19.80
**81**	3′—3″	60	17.85	**85**	7′—7″	60	18.90	**89**	11′—11″	60	22.33
**82**	4′—4″	60	25.42	**86**	8′—8″	80	22.14				

**Table 6:** The information of railway transportation arcs (million ton/year).

*m*	endpoints	*b*_*m*_	*O*_*m*_	*m*	endpoints	*b*_*m*_	*O*_*m*_	*m*	endpoints	*b*_*m*_	*O*_*m*_
**90**	1″ → 2″	90	56.15	**101**	11″ → 3″	90	79.25	**112**	5″ → 9″	90	35.77
**91**	2″ → 1″	90	53.98	**102**	4″ → 5″	90	57.58	**113**	9″ → 5″	90	40.61
**92**	2″ → 3″	90	45.70	**103**	5″ → 4″	90	65.69	**114**	8″ → 10″	90	52.54
**93**	3″ → 2″	90	37.80	**104**	4″ → 6″	90	64.10	**115**	10″ → 8″	90	63.19
**94**	1″ → 4″	90	37.82	**105**	6″ → 4″	90	51.84	**116**	8″ → 11″	90	53.42
**95**	4″ → 1″	90	54.58	**106**	6″ → 5″	90	69.76	**117**	11″ → 8″	90	61.56
**96**	1″ → 7″^a^	50	22.62	**107**	5″ → 6″	90	65.98	**118**	9″ → 10″	90	37.68
**97**	2″ → 8″	100	67.44	**108**	6″ → 7″	90	35.76	**119**	10″ → 9″	90	35.39
**98**	8″ → 2″	100	60.22	**109**	7″ → 6″	90	41.01	**120**	10″ → 11″	90	59.91
**99**	3″ → 8″^b^	50	30.64	**110**	7″ → 8″	90	52.48	**121**	11″ → 10″	90	68.24
**100**	3″ → 11″	90	77.71	**111**	8″ → 7″	90	36.01				

^a,b^ single-track railway

The values of other parameters are shown in Table 7.

**Table 7:** The values of other parameters.

**Parameter**	*e*^road^	*e*^rail^	*θ*	*μ*
**Value**	0.18 CNY/ton-km	0.14 CNY/ton-km	0.01 CNY/h	0.16 PCU/ton
**Parameter**	*v*_*m*_ (∀*m* ∈ *E*^road^**)**	*v*_*m*_ **(**∀*m* ∈ *E*^rail^)	*α*	*β*
**Value**	80 km/h	10.42 km/h	1.5	4

Due to length limitation of the paper, we need to simplify the input data. Thus, we have the following assumptions.

1. The time consumption of departing and arriving of road transportation are 4 hours;
2. The time consumption of departing and arriving of railway transportation are 24 hours and 6 hours respectively;
3. The expenditure of departing and arriving of road transportation are 1 CNY/ton and 0 respectively;
4. The expenditure of departing and arriving of railway transportation are 25.3 CNY/ton and 0 respectively;
5. The expenditure of transferring is 8 CNY/ton and the time consumption is 12 hours;
6. All the railway freight flows go along the shortest path. In this situation, the railway freight flow paths conform to the two rules mentioned before.

### Computational results

We employ Gurobi to solve the problem. The global optimal solution is found within 17.06s. The solution is shown in Table 8.

**Table 8:** The paths and volumes of the shipments.

OD	Path	Volume(million ton/year)
**S05→S01**	S05 → S04 → S01^a^	5.10
**S05→S02**	S05 ⇒ S04 ⇒ S01 ⇒ S02^b^	8.23
**S05→S03**	S05 → S06 → S07 → S08 → S03	3.45
**S06→S01**	S06 → S04 → S01	5.79
	S06 ⇒ S04 ⇒ S01	9.88
**S06→S02**	S06 → S07 → S08 → S02	7.32
	S06 → S07 ⇒ S08 ⇒ S02^c^	5.24
**S06→S03**	S06 → S07 → S08 → S03	13.37
**S07→S01**	S07 → S01	1.02
**S07→S02**	S07 → S08 → S02	5.43
**S07→S03**	S07 → S08 → S03	3.21
**S08→S01**	S08 → S02 ⇒ S01	6.51
**S08→S02**	S08 → S02	4.38
**S08→S03**	S08 → S03	2.56
**S09→S01**	S09 → S05 ⇒ S04 ⇒ S01	6.21
**S09→S02**	S09 ⇒ S10 ⇒ S08 ⇒ S02	11.45
**S09→S03**	S09 → S10 → S08 → S03	0.41
	S09 ⇒ S10 ⇒ S08 ⇒ S03	15.36
**S10→S01**	S10 → S08 ⇒ S02 ⇒ S01	2.38
**S10→S02**	S10 → S08 → S02	1.52
**S10→S03**	S10 → S08 → S03	6.78
**S11→S02**	S11 → S08 → S02	4.21

^a^ → represents that the freight is transported by truck.

^b^ ⇒ represents that the freight is transported by train.

^c^ The underlined node is the node at which the freight is transferred.

According to the statistics of the result, the total bulk freight transport completed by the road network is 42564.04 million ton-kilometers. The total bulk freight transport completed by the railway network is 44222.33 million ton-kilometers. The load (including other vehicles) of the road transportation arcs is shown in Table 9.

**Table 9:** The capacity and load of road transportation arc (million PCU/year).

*m*	Load	*b*_*m*_	*m*	Load	*b*_*m*_	*m*	Load	*b*_*m*_
**1**	24.56	28	**13**	24.12	28	**25**	25.77	28
**2**	25.98	28	**14**	22.54	28	**26**	22.54	28
**3**	24.75	28	**15**	25.42	28	**27**	25.43	28
**4**	22.22	28	**16**	24.58	28	**28**	24.76	28
**5**	25.65	28	**17**	25.34	28	**29**	24.04	28
**6**	23.85	28	**18**	23.37	28	**30**	26.46	28
**7**	25.54	28	**19**	24.33	28	**31**	22.38	28
**8**	22.39	28	**20**	22.77	28	**32**	24.43	28
**9**	24.34	28	**21**	26.82	28	**33**	21.88	28
**10**	27.25	28	**22**	22.44	28	**34**	25.49	28
**11**	28.52	28	**23**	26.47	28			
**12**	22.89	28	**24**	24.77	28			

The load of handling arcs including other categories of freight is shown in Table 10.

**Table 10:** The capacity and load of handling arcs (million ton/year).

*m*	Load	*b*_*m*_	*m*	Load	*b*_*m*_	*m*	Load	*b*_*m*_
**35**	39.82	80	**50**	39.77	80	**65**	41.03	80
**36**	51.48	80	**51**	54.74	80	**66**	59.73	80
**37**	56.92	80	**52**	39.77	80	**67**	44.78	80
**38**	80.00	80	**53**	38.46	80	**68**	44.52	80
**39**	43.67	80	**54**	37.85	80	**69**	61.97	80
**40**	72.04	80	**55**	67.00	90	**70**	39.37	80
**41**	47.26	80	**56**	47.96	90	**71**	45.71	80
**42**	80.00	80	**57**	63.33	80	**72**	41.92	80
**43**	52.28	80	**58**	39.57	80	**73**	45.57	80
**44**	71.71	80	**59**	46.59	80	**74**	51.37	80
**45**	56.47	80	**60**	54.37	80	**75**	55.04	80
**46**	58.20	80	**61**	41.57	80	**76**	53.94	80
**47**	46.67	80	**62**	37.75	80	**77**	47.93	80
**48**	53.37	80	**63**	57.80	80	**78**	44.73	80
**49**	48.96	80	**64**	59.24	80			

The load of transfer arcs including other categories of freight is shown in Table 11.

**Table 11:** The capacity and load of handling arcs and transfer arcs (million ton/year).

*m*	Load	*b*_*m*_	*m*	Load	*b*_*m*_	*m*	Load	*b*_*m*_
**79**	19.99	60	**83**	22.99	60	**87**	20.78	60
**80**	26.72	60	**84**	23.33	60	**88**	19.80	60
**81**	17.85	60	**85**	24.14	60	**89**	22.33	60
**82**	33.65	60	**86**	24.52	80			

The load (including the equivalent freight volume of other trains) of railway arcs are shown in Table 12.

**Table 12:** The capacity and load of railway transportation arcs (million ton/year).

*m*	Load	*b*_*m*_	*m*	Load	*b*_*m*_	*m*	Load	*b*_*m*_
**90**	64.38	90	**101**	79.25	90	**112**	35.77	90
**91**	62.87	90	**102**	57.58	90	**113**	40.61	90
**92**	45.70	90	**103**	71.90	90	**114**	52.54	90
**93**	37.80	90	**104**	64.10	90	**115**	90.00	90
**94**	37.82	90	**105**	61.72	90	**116**	53.42	90
**95**	78.90	90	**106**	69.76	90	**117**	61.56	90
**96**	22.62	50	**107**	65.98	90	**118**	64.49	90
**97**	67.44	100	**108**	35.76	90	**119**	35.39	90
**98**	79.29	100	**109**	41.01	90	**120**	59.91	90
**99**	46.00	50	**110**	57.72	90	**121**	68.24	90
**100**	77.71	90	**111**	36.01	90			

According to the data in Tables 9–12 we can verify the following conclusions.

1. When there is enough capacity, the shipment will go along the path of the lowest general cost;
2. When the capacities of some sections are insufficient, some shipments will go along the non-shortest path. In this situation, the model assigns the freight flow based on the principle of system optimization.

The above conclusions are consensus with reality.

### Sensitivity analysis

**Fig 7:**
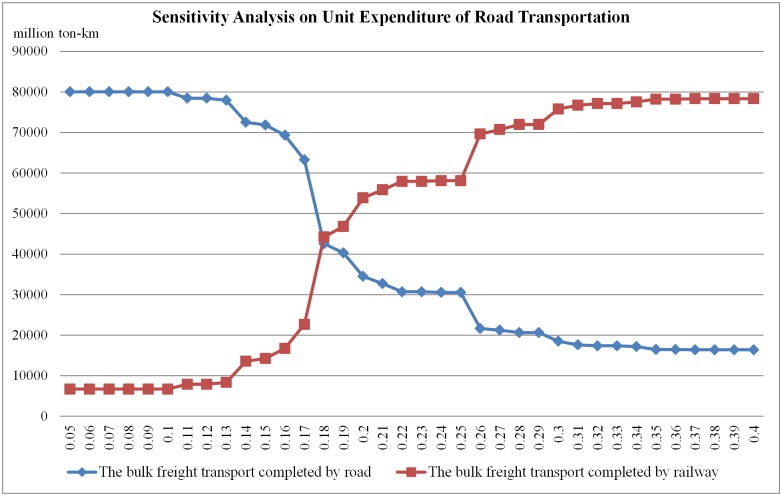
Illustration of sensitivity analysis on unit expenditure of road transportation.

Since the model is a tool to analyze the measures for diverting freight flow from road to railway and most measures such as tax, discount etc. will influence the monetary expenditure of shippers. Analyzing the sensitivity of the expenditure parameter in the model is an effective way to estimate the measures. Therefore, we change the expenditures of road and railway in certain ranges and analyze how the loads on road and railway changes. First, we set the unit expenditure of railway transportation as 0.14 CNY/ton-km and change the unit expenditure of road transportation from 0.05 CNY/ton-km to 0.40 CNY/ton-km by the step of 0.01 CNY/ton-km. The load on road and railway is shown in Fig 7.

From Fig 7 we can know that the unit expenditure of road transportation will influence the freight flow assignment between road and railway significantly when it is in the range from 0.13 CNY/ton-km to 0.26 CNY/ton-km. In other words, the share of freight flow between road and railway is sensitive to the unit expenditure of road transportation in this situation. When the expenditure is out of the range, the measure of adjusting the expenditure will generate little effect because of other restrictions such as the capacity of the network.

**Fig 8:**
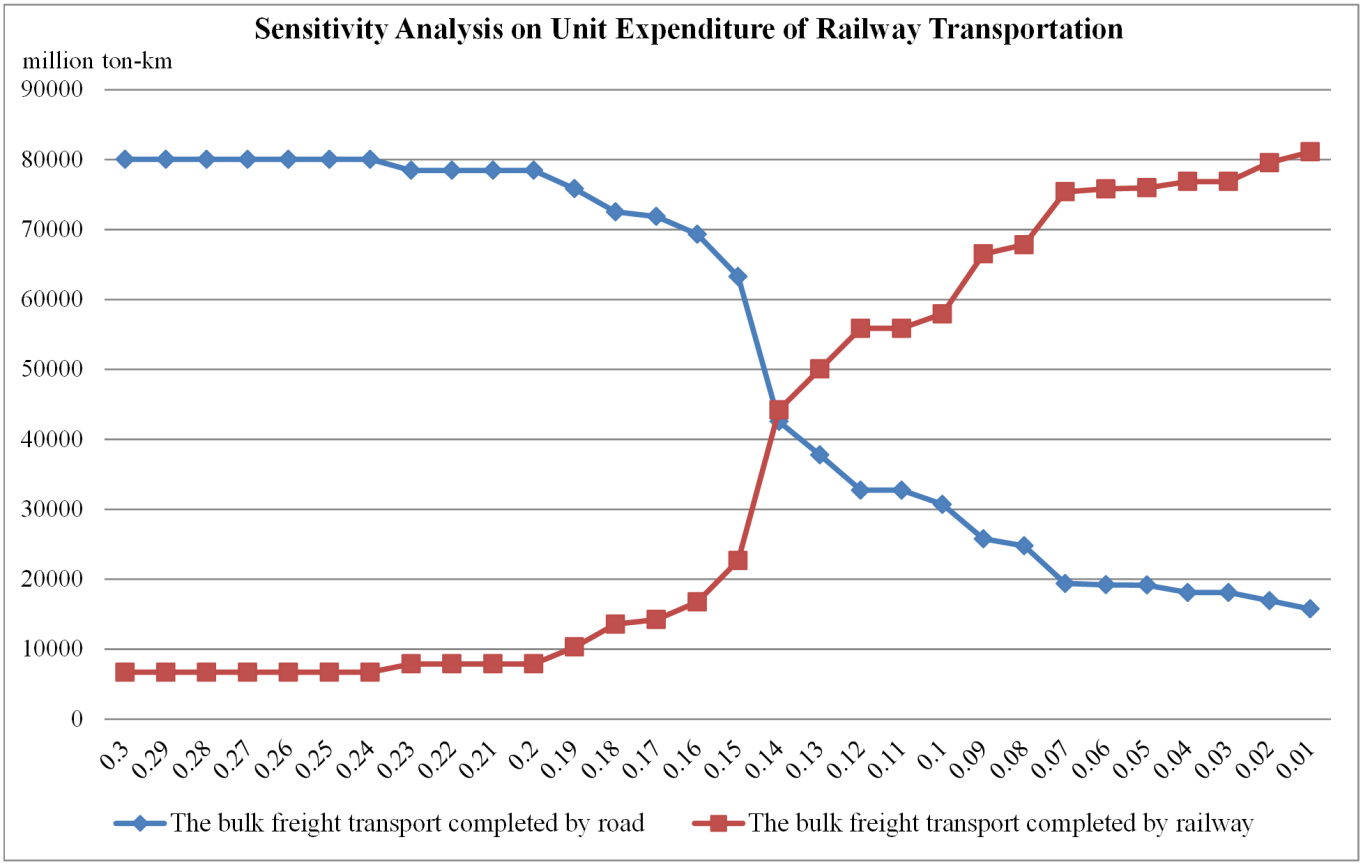
Illustration of sensitivity analysis on unit expenditure of railway transportation.

Then we set the unit expenditure of road transportation as 0.18 CNY/ton-km and change the unit expenditure of railway transportation from 0.30 CNY/ton-km to 0.01 CNY/ton-km by the step of 0.01 CNY/ton-km. The load on road and railway is shown in Fig 8.

From Fig 8 we can know that the unit expenditure of railway transportation will influence the freight flow assignment between road and railway significantly when it is in the range from 0.20 CNY/ton-km to 0.07 CNY/ton-km. In other words, the share of freight flow between road and railway is sensitive to the unit expenditure of railway transportation in this situation. When the expenditure is out of the range, the measure of adjusting the expenditure will generate little effect because of other restrictions such as the capacity of the network.

In practical work, by using the data from reality, the influence of road and railway costs on the assignment of freight flow on road and railway can be analyzed quantitatively. The analysis will provide valuable reference for designing the strategy of diverting freight flow from road to railway.

## Conclusion

Reducing the carbon emissions of inland transportation system by optimizing the share of freight flow between road and railway becomes an important issue for developing low-carbon transportation. In order to design proper measures for adjusting fright flow between road and railway, the distribution of freight flow on the road and railway network under certain situations and the factors which influence the distribution should be acquainted. Therefore, this paper mainly focuses on the freight flow assignment model for simulating the flow distribution on road and railway network. Firstly, the factors which influence the decision making of shippers are analyzed and the basic principle of the freight flow assignment is proposed. Then a “general network” which contains transportation, handling and transferring is established and general cost functions which are used to describe the weight of the arcs in the network are formulated. The functions take the characters of road transportation, railway transportation, freight handling and transferring into consideration to make the model more practical. Based on this, a user equilibrium model for freight flow assignment on “general network” is formulated and the features of the model are analyzed. In the stage of model preprocessing, a new method for generating the available paths on the “general network” is put forward. The method considers the characters of railway freight flow path and road freight flow path respectively. In terms of the solving method for the model, since the model is a nonlinear one which is difficult to solve by traditional algorithm, a linearizing method is adopted to convert the model into a linear one. Thus the model is easy to solve with little error. Finally, a numerical example is made to test the rationality and feasibility of the model. In the example, sensitivity analysis on the expenditure of road and railway transportation is made to reveal their quantitative influence on the flow distribution. This provides theoretical method for estimating the measures for diverting bulk freight flow from road to railway.
